# Screening for viral pathogens in the gastrointestinal tract from cases of sudden unexpected death in infancy at the Tygerberg Medico-legal Mortuary

**DOI:** 10.1186/s12985-023-02249-y

**Published:** 2023-11-29

**Authors:** Danielle T Cupido, Corena de Beer

**Affiliations:** Division of Medical Virology, Department of Pathology, Stellenbosch University, PO Box 241, Cape Town, 8000 South Africa

**Keywords:** Viral gastroenteritis, Sudden unexpected death in infancy, SUDI, Viral enteropathogens

## Abstract

Sudden and unexpected death in infancy (SUDI) may be triggered by an external risk or exposure. Intestinal infections with enteric viruses may disrupt the gut and enhance bacterial toxins present in SUDI cases. While diarrhoeal disease deaths have decreased worldwide, approximately half a million deaths still occur in children in Sub- Saharan Africa and South Asia. Furthermore, the role of viral enteropathogens in SUDI cases have not been investigated. The aim of this study was to describe specific viral pathogens in stool samples collected from SUDI cases and age-matched, apparently healthy infants in Cape Town, South Africa. Stool samples were collected from 176 SUDI cases between June 2017 and May 2018. In addition, stool samples were collected from the nappies of 30 age-matched, apparently healthy infants as a control group. Real-time polymerase chain reaction was performed on the stool samples for viral detection. A total of 111 SUDI cases were positive for viruses, with rotavirus (38.6%; 68/176) and norovirus GI and GII (30.0%; 53/176) were prevalent in SUDI cases. Adenovirus Type F was present in only 15.9% (28/176), astrovirus in 9.7% (17/176), and sapovirus in 0.6% (1/176) of cases. In the control samples, norovirus GII was detected most frequently (36.7%; 11/30), followed by rotavirus (33.3%; 10/30), and sapovirus in 6.7% (2/30). While there was no significant association between SUDI cases and enteric viruses, the majority of viruses were significantly associated with the seasons. The study confirms the importance of rotavirus vaccination and describes the significance of norovirus infection in children, post rotavirus vaccine introduction.

**Supplementary Information:**

The online version contains supplementary material available at 10.1186/s12985-023-02249-y.

## Introduction

The sudden and unexpected death of seemingly healthy infants has been recognised since ancient times [1]. Sudden and unexpected death in infancy (SUDI) is not a clinical or pathological diagnosis and is different from sudden infant death syndrome (SIDS). SUDI includes all deaths in infants younger than 1 year (often restricted to 7-365 days) that present suddenly and unexpectedly [2], or at least without a clearly identifiable cause at the time of death and *before* any investigations have been performed. In contrast, SIDS is defined as the unexpected death of an infant under the age of one, usually occurring during sleep, where no explanation can be found after a thorough investigation has been conducted. This includes an autopsy and an examination of the circumstances of death and medical history [3].

SUDI occurs when infants with latent vulnerabilities are exposed to a trigger event or extrinsic risk factor during a critical developmental phase [4]. If protective mechanisms are ineffective during these episodes, SUDI will be more likely to occur [5, 6]. Extrinsic risk factors include sleeping in prone position, bedsharing, overheating, using soft bedding, sleeping on inappropriate surfaces, and covering the face of an infant while sleeping. Infants are more vulnerable to the influences of extrinsic risks due to intrinsic risk factors [7].

Viruses can enhance the noxiousness of bacterial toxins in SUDI [8]. Viral diarrhoea is caused by enteric viruses colonising different environments of the small intestine, disrupting the natural fluid balance of the gut [9]. A global decline in the number of deaths due to diarrhoeal disease has occurred, with approximately half a million of those deaths occurring in children [10]. Sub-Saharan Africa and South Asia account for most diarrhoeal deaths, with acute gastroenteritis accounting for up to 10% of hospitalisations [11] and 19% of deaths in children under five years [12]. The majority of gastroenteritis cases globally are caused by enteric viruses [13], with over 20 confirmed aetiological agents [14]. Dehydration, electrolyte disturbance, and metabolic acidosis are common complications, with the risk increasing in children with poor nourishment [15].

Rotavirus (RV) is the most common viral enteropathogen in infants and young children, particularly in acute diarrhoea [16]. In 2019, 151 714 children under five died from RV infection [17], with over 200 000 deaths in sub-Saharan Africa and Southeast Asia [18]. The RV vaccine has significantly reduced RV-specific and all-cause diarrhoea [19], with the South African Extended Programme for Immunisation (EPI) introducing it in 2009 (National Department of Health, 2012). Norovirus (NoV) is also a major contributor to severe viral gastroenteritis [20, 21], with most cases caused by NoV genogroup II strains [22]. NoV vaccine development is a high priority, but challenges remain due to genetic and antigenic diversity [23]. Clinical trials are underway for recombinant VP1-based virus-like particles (VLPs), which mimic major antigens from NoVs and are safe, immunogenic, and protective [24].

Sapovirus (SaV) has become a major viral enteropathogen in countries with high RV vaccination coverage [25], with prevalence rates in children under five years of age varying across sub-Saharan African countries [26–30]. SaV is associated with both sporadic and epidemic cases of acute gastroenteritis, with children being more affected than adults [31–33]. Research on SaV is limited [34], but it has been detected in a few cases. Astrovirus (ASV) commonly causes diarrhoea in children, the elderly, and immunocompromised individuals [35–39]. In 2016, human adenovirus (AdV) was responsible for about 13% of all deaths in children under five globally [40]. Gastroenteritis associated with AdV is commonly caused by types 40 and 41 [41–44]. Post-mortem evidence suggests that infection is a leading contender in terms of causation of SUDI as cases often show coliform bacterial colonisation in the respiratory tract [45–47] and toxigenic organisms in the gastrointestinal tract (GIT) such as Staphylococcus aureus (S. aureus) [48]. Often, SUDI cases have mild clinical symptoms during the days prior to death, with diarrhoea often being reported [49].

To date, no studies have investigated the role of viral enteropathogens in SUDI cases. This study therefore aimed to describe specific viral pathogens in stool samples collected from SUDI cases and age-matched, clinically healthy infants in Cape Town, South Africa.

## Materials and methods

### Sample collection

Stool samples were collected from 176 SUDI cases admitted to the Tygerberg Medico-legal Mortuary, Cape Town, between June 2017 and May 2018. Only infants aged between seven days and one year who died suddenly and unexpectedly without a pre-existing medical condition or apparent cause met the criteria for SUDI and were included. To serve as a control group, stool samples were also collected non-invasively from the nappies of 30 age-matched, apparently healthy infants between May 2018 and December 2019. Convenience sampling was used for the control group.

### Nucleic acid extraction

Stool samples were collected in sterile leakproof containers and stored in DNA/RNA Shield™ (Zymo Research, California, USA) at -80 °C until analysis. Viral DNA and RNA were extracted using NucleoSpin Virus Kit (Macherey-Nagel, Düren, Germany) according to the manufacturer’s instructions. The quantity and purity of the extracted DNA/RNA were determined using the NanoDrop ND-1000 spectrophotometer (ThermoFisher Scientific, USA).

### Qualitative real-time polymerase chain reaction for enteric viruses

Multiplex polymerase chain reaction (PCR) using the Allplex ™ GI-Viral Assay (Seegene Inc, Korea) was performed following the manufacturer’s instructions for detection of NoV GI, NoV GII, RVA, AdV-F (Serotype 40/41), ASV and SaV (Genogroups G1, 2, 4) in human stool specimens.

### Detection of diarrhoeagenic *E. coli*

Swabs and stool samples collected from the SUDI cases were sent to the National Health Laboratory Services (NHLS), Tygerberg Hospital, microbiology laboratory for routine identification of organisms. *Escherichia coli* (*E. coli*) was confirmed to be present in the samples by inoculating them onto MacConkey agar plates. Deoxyribonucleic acid (DNA) was extracted from the *E. coli* using a Zymo Quick-DNA™ Miniprep Plus Kit (Zymo Research, USA) according to the manufacturer’s instructions. The Allplex™ GI-Bacteria (II) Assay (Seegene Inc, Korea) was performed on a CFX96 system (Bio-Rad, USA) according to the manufacturer’s instructions to detect genes for EHEC (stx1/stx2, *E. coli* O157), EPEC (eaeA), ETEC (lt/st) and EAEC (aggR) and internal control.

### Statistical analysis

Statistica® version 14.0 Statistical Software (TIBCO Software, Inc.) was used to perform analyses. The viruses detected in the SUDI cases were correlated with the risk factors associated with SUDI. Associations were calculated using the Mann-Whitney U test for numerical data and the Fisher exact test for categorical data. Statistical significance was observed at p < 0.05 at a 95% confidence interval, with a strong significance observed at p < 0.01. In the case of non-normally distributed data, the Mann-Whitney U and Fisher exact tests were used.

### Ethics

The study was approved by the Stellenbosch University Health Research Ethics Committee (S16/10/214) and a waiver of consent was granted for the SUDI cases. Informed consent was obtained from the parents of the infants included in the control group.

## Results

### Sociodemographic data

A total of 176 cases (57.4% (101/176) males and 42.6% (75/176) females) were included in the study group. The median age of the infants at the time of death was 8.3 weeks (interquartile range [IQR]: 4.8–16.6), and the mean post-mortem interval (PMI) was 6.6 ± 3.5 days.

Risk factors reported most frequently were bed-sharing (84%, 148/176), side sleeping position (49%, 86/176), informal housing (44%, 78/176) and prematurity (39%, 69/176). The majority of cases (65%, 115/176) presented during the colder months of the year (April to August) compared to the warmer months (September to March).

The control group comprised 40% (12/30) males and 60% (18/30) females, with a median age of 24 weeks (IQR: 9–38). Bed-sharing was reported in 20% (6/30) of the controls, with infants placed to in the side position in 47% (14/30), and 20% (6/30) of the infants were born prematurely.

### Laboratory results

A total of 111 SUDI cases were positive for viruses, while no viruses were identified in the remaining 65 cases. A single virus was detected in 63 SUDI cases and 17 controls. NoV GII was detected in 11 samples, while 10 were negative for all viruses (Table 1). Among the enteric viruses detected, 36 cases had co-detections of two viruses, while three controls had co-detections of two viruses (Table 2). Viral and bacterial co-detection was found in 83 (47%) cases. The most prevalent co-detections were one virus and two or more bacteria (Table 3). The most prevalent viral and bacterial co-detection patterns were NoV (GI/GII) and diarrhoeagenic E. coli (DEC) in 21cases (25.3%) as well as RVA and DEC in 21cases (25.3%).

**Table 1:** Frequency (n, %) of GIT viruses detected in 176 SUDI cases and 30 control samples

GIT viruses	SUDI Cases (n = 176)	Controls (n = 30)
RVA	68 (38.6%)	10 (33.3%)
NoV	53 (30%)	11 (36.7%)
AdV-F	28 (15.9%)	0 (0%)
ASV	17 (9.7%)	0 (0%)
SaV	1 (0.6%)	2 (6.7%)
Negative	65 (36.9%)	10 (33.3%)

**Table 2:** Prevalence of multiple pathogens detected in the cases and control group

No. of viruses	Virus combinations	SUDI Cases (n = 48)	Controls (n = 3)
Two	NoV GII + RVA AdV-F + RVA NoV GII + AdV-F ASV + RVA NoV GI + RVA ASV + SaV NoV GII + NoV GI Double infections in total (%)	8 8 3 13 1 1 2 36 (75%)	3 0 0 0 0 0 0 3 (100%)
Three	NoV GII + AdV-F + RVA NoV GII + AdV-F + NoV GI Triple infections in total (%)	10 1 11 (22.9%)	0 0 0 (0%)
Four	ASV + NoV GII + NoV GI + RVA Quadruple infections in total (%)	1 1 (2.1%)	0 0 (0%)

**Table 3:** Prevalence of viral and bacterial co-detection in the cases (n = 176)

Viral and bacterial co-detection	Viral and bacterial combinations	SUDI cases
One virus + two or more bacteria	NoV (GI/GII) + DEC RV-A + DEC ADV-F + DEC Total co-infections (%)	21 21 4 46 (26.1%)
Two viruses + two or more bacteria	ASV + RV-A + DEC ADV-F + RV-A + DEC NoV (GI/GII) + RV-A + DEC NoV (GI/GII) + ADV-F + DEC Total co-infections (%)	7 5 5 2 19 (10.8%)
Three viruses + two or more bacteria	NoV (GI/GII) + ADV-F + RV-A + DEC Total co-infections (%)	6 6 (3.4%)
One virus + one bacterium	NoV (GI/GII) + DEC RV-A + DEC ADV-F + DEC Total co-infections (%)	3 1 1 5 (2.8.7%)
Three viruses + one bacterium	NoV (GI/GII) + ADV-F + RV-A + DEC Total co-infections (%)	3 3 (1.7%)
Two viruses + one bacterium	NoV (GI/GII) + DEC ADV-F + DEC Total co-infections (%)	2 1 3 (1.7%)

**Fig. 1:**
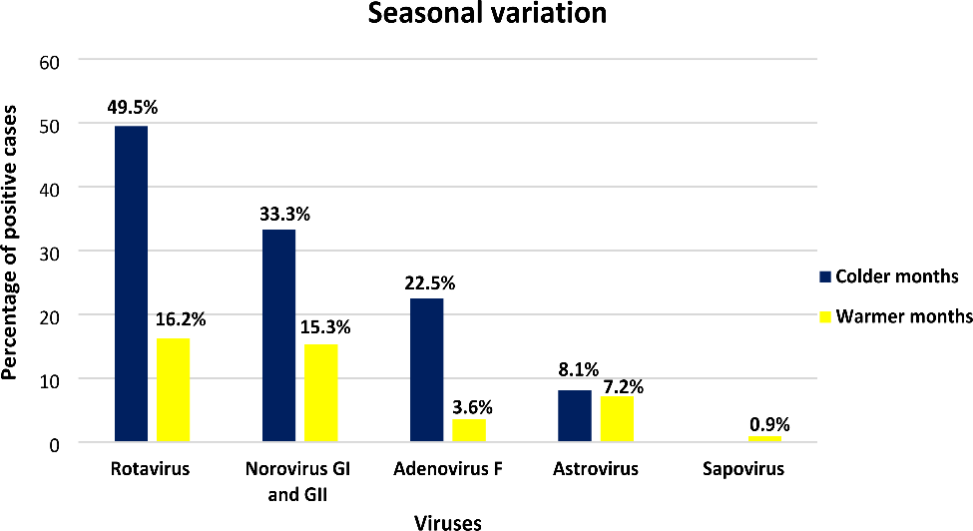
Seasonal variation of enteric viruses detected in the SUDI cases from June 2017 to May 2018

Seasonal variations in the occurrence of enteric viruses were evident (Fig. 1). RVA, NoV GI, NoV GII, AdV-F and ASV were more frequently detected in the colder months (49.5%, 33.3%, 22.5% and 8.1% respectively) compared to the warmer months, with SaV the least frequently detected (0.9%).

Histopathology was conducted on tissues specimens from the gastrointestinal tract of SUDI cases but did not provide any significant results due to autolysis of most slides (Supplementary data).

### Statistical analysis

In the SUDI group, a highly significant association was observed between RVA, NoV GII, AdV-F, ASV and month of death (Fisher exact test, p < 0.01). These four viruses also showed a significant association with seasons. NoV GI and SaV displayed no association with month of death or seasons. None of the viruses were associated with sex, prematurity, bed-sharing, ventilation, PMI and number of people in the household. AdV-F showed significant association with both age in weeks and birthweight (Mann-Whitney U test, p = 0.01), while NoV GI had a significant association with sleeping position (Fisher Exact test, p = 0.03) and medical history prior to death (Fisher exact test, p = 0.04).

## Discussion

Globally, enteric viruses have been recognised as the most common cause of gastroenteritis [50]. In the current study, enteric viruses were detected in more than 60% of both SUDI cases and controls, which is similar to data from Gabon [26] but considerably lower than Burkina Faso [42]. In contrast, lower prevalence rates between 30% and 54% were found in Cameroon [51], India [52], Nigeria [53], Europe [54] and Italy [55]. It is therefore apparent that the viral gastroenteritis burden varies among locations and countries, but it is clear that low- and middle‐income countries are affected disproportionately [53].

Rotavirus A was the prominent pathogen detected in the SUDI cases. Previous South African studies described the distribution and diversity of enteric viruses in stool samples collected from children younger than five years [56, 57], as well as the possible mixed diarrhoeal aetiology in stool samples collected from children younger than four years [58]. Rotavirus A was identified as the predominant enteric virus in 22% of children [56, 57] and in 82% of infants under 12 months old [58]. In low- and middle-income countries such as South Africa, RV is still the primary cause of viral gastroenteritis hospitalisations, in spite of routine vaccination programs [59].

Norovirus, predominantly NoV GII, was the second-most frequently detected virus in the SUDI cases and the most frequent virus in the control group. Similarly, genogroup NoV GII has been found in clinical cases and disseminated in communities globally [60–62]. Generally, children living in areas with poor sanitation and hygiene practices are at increased risk of exposure to enteropathogens, which, similar to other middle-income countries, such as Mexico, Brazil, Bolivia and China, could be associated with higher NoV GII prevalence rates [63–66].

The low prevalence of AdV-F infection found in this study is marginally higher than in previous South African studies that found AdV in 7–12% of cases [67, 56, 57]. The marginally higher prevalence rate is likely to result from the virus being more endemic in the sampling region in comparison to the other study regions. In contrast, a study from Ethiopia describing the prevalence and genetic diversity of human AdV and human ASV in stool samples collected from infants and children with diarrhoea found a much higher AdV prevalence (32%) [68]. The lower prevalence in the current study may be the result of the use of a less sensitive assay than that used in the study in Ethiopia, which was more sensitive and able to detect all known types of human AdV.

Astrovirus usually causes milder infections that do not require hospitalisation [37]. A study from Kenya and Gambia investigated the prevalence and diversity of both classic and novel ASV in children under five and confirmed the presence of ASV in 10% of cases [69]. However, a Nigerian study examining the prevalence, seasonality and risk factors of enteric viruses in stool samples of children with acute gastroenteritis found ASV in 20% of these cases [53]). In contrast to the current study, a retrospective South African study [70] screened diarrhoeagenic stool samples from children under the age of five for viruses, bacteria and parasites and found ASV in only about 6% of infants who died during the course of the study. Infants in the current study died suddenly and unexpectedly before hospitalisation could possibly occur, whereas those in the retrospective study were hospitalised for an average of four days before passing away. This may explain the higher prevalence observed. Furthermore, it is likely that the higher prevalence rate may be due to the fact that ASV infection is frequently associated with the lack of a source of indoor water, and contaminated water sources such as boreholes, cisterns, communal taps, and rivers. [71].

Although SaV was the least prevalent virus in the cases in this study, found in less than 1%, the prevalence found in the control group was comparable to the 8% found in hospitalised children under five years old with acute diarrhoea between 2009 and 2013 [72]. In comparison to RV or NoV, SaV can present with a rather mild clinical presentation [73, 74], and although mortality associated with SaV is rare, outbreaks have been reported in elderly long-term care facilities [34]. This may explain the higher prevalence in the control group than in the cases. The Malnutrition and Enteric Disease Study (MAL-ED) reported a 23% SaV detection rate globally, including South Africa [32]. Different prevalence rates amid distinct studies are common and may possibly be linked to different study designs, study settings, sample sizes, sampling seasons, socioeconomic status of the population and viral detection procedures used during the investigations [75].

Almost half of the current cases had co-detections, which was about 10% more than a previous study of South African children under five years of age hospitalised for gastroenteritis [56, 57]. Similarly, a study in Burkina Faso reported enteric viruses in 35% of children under five years of age [42]. The most commonly reported co-detection in this study was between ASV and RVA, which is in agreement with previous findings from South Africa [56, 57]. The prevalence rate was, however, much lower than reports from Nigeria (59%) [53]. This study also found co-detections between NoV GII, RVA and AdV more frequently than results from Kuwait (21% versus 8%) [76]. There are biological and epidemiological implications of mixed viral infections whereby viruses interact either synergistically or antagonistically, changing the concentration of either or both viruses and impacting the outcome of the infection [76].

Co-detection between bacteria and viruses was confirmed in 47% of cases. Similarly, a study conducted in South Africa to determine the prevalence of individual and multiple diarrhoea-causing pathogen combinations among children suffering from diarrhoea in rural and peri urban communities reported a 47% prevalence of bacterial and viral co-infection [77]. The prevalence of co-detection between NoV (GI/GII) and DEC as well as RV-A and DEC in this study was higher than that in Southwest China (25.3% versus 1.1% and 25.3% versus 2.3%, respectively) [78]. The effects of co-infections on the intestinal flora have been shown to alter its composition, reduce its diversity, and increase the frequency of intestinal flora disorders [79, 80]. Inflammatory processes may be triggered by microbiota that directly interact with epithelial cells [81].

The lack of histopathology results from the GIT may be due to the abundance of self-digesting enzymes in this tissue type, leading to autolysis [82]. Compared with histology slides of other organs, such as the lung and heart, which are routinely examined, it was difficult to analyse the GIT slides in this study due toPMI and autolysis. As a longer PMI has the effect of deteriorating the quality of the tissues and consequently, the quality of the results. [83].

Colder months (April to August) have repeatedly been associated with a spike in infant deaths compared to warmer months (September to March) [84]. Different mechanisms may be at play. Cold weather increases the likelihood of close contact among people who may have been exposed to fomites contaminated with viruses, thereby increasing the risk of transmission from person to person [53]. These conditions may contribute to the organism’s spreading, transmitting, and maintaining itself [85], which was supported by the incidence of viral peaks during the colder months in this study.

The highest incidence of SUDI has repeatedly been shown to occur in early infancy (2–4 months) when immune responses are still immature and levels of maternal antibodies are declining [86]. Birth weight below 2 500 g is also associated with a higher risk of infection [87] as well as impaired homeostasis, which can lead to infection [88]. This supports the associations observed between age and ADV-F as well as birth weight. As opposed to other GIT viruses, AdV infections can be contracted throughout the year, which may explain the statistical associations observed, especially among infants with compromised immune systems.

An association was observed between NoV GI and GII and sleeping position as well as the medical history of the infant prior to death. It has been reported in a study conducted in Tasmania that SIDS can occur in the prone position if accompanying symptoms, such as cough, fever, nasal congestion, vomiting, or diarrhoea, are present on the day of death or the day prior. Among infants in the prone position, the risk of SIDS was higher for those who were ill than for those who were healthy [89]. This could explain the association observed in the current study.

The major limitation of this study was the small number of controls included. A suitable control group would be infants who died from unnatural causes, but several factors and ethical constraints prevented this, as samples of these infants were not used to determine the cause of death, and as a result, are not covered by the waiver of consent. Samples could be collected from healthy babies as they represent the closest control group. However, bias was introduced by the small ample size and use of convenience sampling, which resulted in an unequal sample size and age-distribution between the cases and controls. The medical history of the SUDI cases, i.e., whether they had diarrhoea or any other clinical symptoms before death, was not always available or subject to recall bias when the parents completed the questionnaire upon admission of the infant to the Tygerberg Medico-legal Mortuary.

## Conclusion

Based on the results of this study, RVA and NoV were identified as the most common enteric viruses in infants under 12 months of age. It is imperative to highlight both the importance of RVA vaccinations and the significance of NoV infection in children following RV vaccination. It is particularly critical in a middle-income country such as South Africa where infections caused by RVA remain the leading cause of viral gastroenteritis despite routine vaccinations. It has been demonstrated that co-infection with multiple microbes can increase both morbidity and mortality. The identification of pathogens in infants with diarrhoea is therefore imperative. It was found that while there was no significant association between SUDI cases and enteric viruses, the majority of viruses were significantly associated with the seasons. Colder months have repeatedly been associated with a spike in infant deaths compared to warmer months. This finding also supports the theory that colder weather provides an ideal condition for viruses to persist and spread.

### Electronic supplementary material

Below is the link to the electronic supplementary material.

Supplementary Material 1: The materials and methods used for the histological analysis. Table S1: Histology results observed in the GIT sections for the SUDI cases

## Data Availability

Access to the data may be obtained by contacting the corresponding author.
