# Supplementary material for: Screening for viral pathogens in the gastrointestinal tract from cases of sudden unexpected death in infancy at the Tygerberg Medico-legal Mortuary
Source: Virol J. 2023 Nov 29;20:281. doi: 10.1186/s12985-023-02249-y (PMC10688011; doi:10.1186/s12985-023-02249-y)
Supplement: Supplementary file 1 — Supplementary Material 1: The materials and methods used for the histological analysis. Table S1: Histology results observed in the GIT sections for the SUDI cases [file 12985_2023_2249_MOESM1_ESM.docx]

Materials and methods

Histology

Tissue samples from the GIT (duodenum, small and large bowel) were collected for histological analysis and placed in Tissue-Tek Uni-Cassettes and fixed in 10% formalin for up to 24 hours to harden and preserve the tissue. Following preservation, the preserved tissue was processed using the Tissue-Tek^®^ VIPTM 5 Vacuum Infiltrator Processor (Sakura^®^ Finetek, Europe). After 12-24 hours fixation in formalin, the tissue was dehydrated and submersed in xylene, followed by embedding the processed tissue in paraffin wax blocks. The embedded tissue was cut into 3-5 µm thick sections using an Accu-Cut^®^ SRMTM microtome (Sakura^®^ Finetek, Europe). The wax sections were placed in a AWB 210 Water Bath (Amos Scientific, Australia) at 60°C to remove folds, mounted on glass microscope slides (Starfrost^®^, UK) and incubated (Scientific Series 9000) for 30 minutes at 77°C. The tissue was stained with haematoxylin and eosin (H&E) (Sigma-Aldrich, South Africa) for visualisation under light microscope (Titford, 2005) The H&E staining technique allowed identification of the morphological changes within GIT tissue. An Olympus^®^ BX41 light microscope was used to analyse the stained microscope slides at magnifications of 40x, 100x and 200x for features such as lymphocytic and neutrophil infiltrates, oedema and inflammation.

Results

Histopathological analysis did not provide any significant results. Autolysis was prevalent on most slides. It was not possible to determine whether autolysis could have obscured any morphological changes. The following example illustrates the histology results of this study (Table 3).

Table S1: Histology results observed in the GIT sections for the SUDI cases.

| SUDI cases | Histology |
| --- | --- |
| 0034/2018 | No significant findings |
| 0082/2018 | Autolysis |
| 0124/2018 | No significant findings |
| 0152/2018 | No significant findings |
| 0174/2018 | Autolysis |
| 0238/2018 | No significant findings |
| 0374/2018 | Autolysis |
| 0540/2018 | Autolysis |
| 0846/2018 | Autolysis |
| 0921/2018 | No significant findings |
| 0947/2018 | Autolysis |
| 0952/2018 | Autolysis |
| 1123/2018 | Autolysis |
